# Radiographic Evidence of Sufficient Transverse Plane Alignment after Weil Osteotomy without Screw Fixation

**DOI:** 10.3390/jcm13020331

**Published:** 2024-01-06

**Authors:** Leona Marleen Ram, Philipp Schippers, Oliver Neun, Yves Gramlich, Eva Herrmann, Alexander Klug, Reinhard Hoffmann, Sebastian Fischer

**Affiliations:** 1 Department for Trauma and Orthopaedic Surgery, Berufsgenossenschaftliche Unfallklinik Frankfurt am Main, 60389 Frankfurt, Germany; 2 Department of Orthopedics and Traumatology, University Medical Center of the Johannes Gutenberg, University Mainz, 55131 Mainz, Germany; 3 Department of Foot and Ankle Surgery, Berufsgenossenschaftliche Unfallklinik Frankfurt am Main, 60389 Frankfurt, Germany; 4 Institut für Biostatistik und Mathematische Modellierung, Goethe-Universität Frankfurt am Main, Theodor-Stern-Kai 7, 60596 Frankfurt am Main, Germany

**Keywords:** Weil osteotomy, metatarsalgia, metatarsophalangeal angle, transversal plane alignment

## Abstract

Weil osteotomy is a proven procedure to restore the harmonic distal parabola of the forefoot. In addition to the proximal displacement of the head in the sagittal plane, a displacement in the transverse plane may be necessary, with the refixation of the displaced metatarsal head historically performed by screw fixation. We aimed to determine the radiological differences among 136 feet of 127 patients with 256 Weil osteotomies retrospectively enrolled and divided into groups with (n = 182) and without (n = 74) screw fixation. Demographic data, radiographic union, pre- and postoperative metatarsal angles, and differences in the dorsoplantar view were evaluated. The mean follow-up period was 3.6 months. The mean preoperative metatarsophalangeal angle was 9.24°, and the mean postoperative angle was 12.99°. The restoration of the transversal alignment plane was equally successful in both groups, with a mean extent of angle correction of 10.58°. No nonunions of the osteotomized metatarsals were observed. The radiographic comparisons revealed no significant difference between the groups (*p* > 0.05). However, visibility of the joint space of the metatarsophalangeal joint was achieved significantly more often in the group without screw fixation (*p* < 0.05). In the absence of bony malunion and the satisfactory restoration of a harmonious parabola of the forefoot, apparently there does not appear to be a necessity for regular screw fixation after Weil osteotomy based on the available data from the present study.

## 1. Introduction

Weil osteotomy has been used regularly since the early 1990s to address painful metatarsalgia [1]. Although clinical outcomes are affected by a high rate of floating toes, this procedure provides very satisfactory results with regard to the original indication [2]. Metatarsalgia, medial and lateral deviation of the lesser toes, and hammer toe deformity are largely typical in old age. Although typical for older people, such a deformity represents an impairment in daily life. This restriction not only results in painful shoe conflicts but also an increased risk of falling due to altered gait patterns. Based on this realization, the number of surgical treatments is increasing. Weil osteotomy has now been established to correct not only metatarsalgia with increased plantar contact pressure but also flexible claw toes and lateral deviations with a disturbed parabola of the forefoot. Usually, the fixation of the metatarsal head after subcapital osteotomy is performed with a screw. In this way, the metatarsal head can be placed and permanently fixed in the sagittal as well as the transverse planes according to the preoperative planning and/or the intraoperative findings. In line with the general endeavor to develop innovative implants and, above all, surgical techniques, the minimally invasive procedure has also become established in the treatment of deformities of the forefoot. Distal metatarsal minimal-invasive osteotomy (DMMO) not only enables a gentle procedure but also promotes the possibility of getting by without osteosynthesis. It should be noted that the terms percutaneous, minimally invasive, mini-open, and open are not uniformly defined. The only certain difference is that with the percutaneous or minimally invasive approach, a milling machine is usually used instead of a saw for the osteotomy. A comparison of DMMO with and without screw fixation confirmed both procedures to be equally effective [3,4]. 

**Figure 1:**
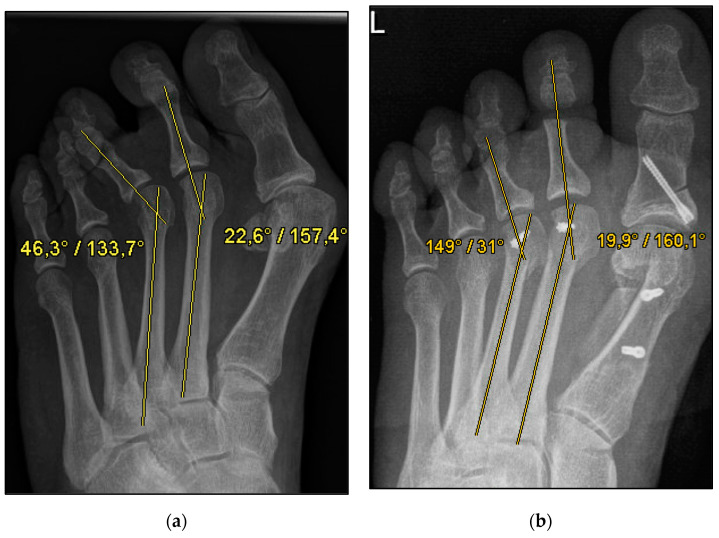
Pre- and postoperative radiographic results of combined hallux valgus surgery and Weil osteotomy of metatarsal 2 and 3 (group A with screw), left foot. (**a**) Weight-bearing radiograph: anteroposterior view preoperative, (**b**) weight-bearing radiograph: anteroposterior view, 6 weeks postoperative.

**Figure 2:**
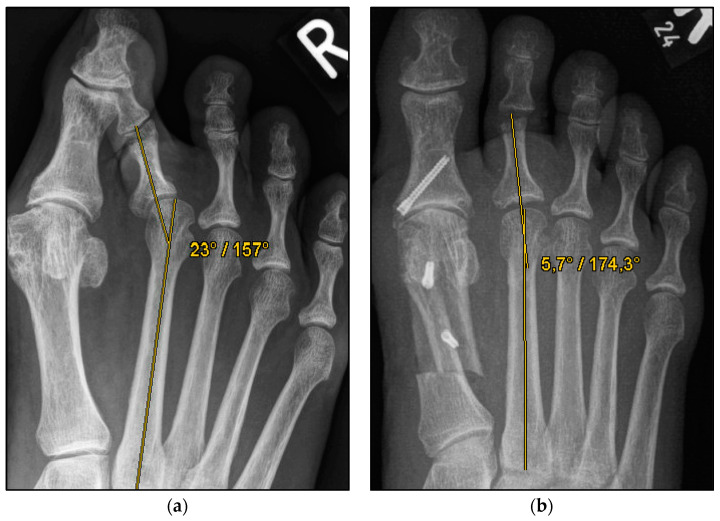
Pre- and postoperative radiographic results of combined hallux valgus surgery and Weil osteotomy of metatarsal 2 (group B without screw), right foot. (**a**) Weight-bearing radiograph: anteroposterior view preoperative, (**b**) weight-bearing radiograph: anteroposterior view, 2 months postoperative.

We aimed to show that conventional open Weil osteotomy has an equally high correction potential regarding transverse plane alignment, even without screw fixation. (Figure 1 and Figure 2) For this purpose, the pre-, intra-, and weight-bearing postoperative radiographs after Weil osteotomy with and without screw fixation were compared. In addition, the radiographs were evaluated with regard to the metatarsophalangeal angle, malalignment, and delayed union and nonunion. This radiological study explicitly aimed to reveal a possible loss of correction after the bony union. The clinical results will be evaluated separately.

## 2. Materials and Methods

### 2.1. Population

**Figure 3:**
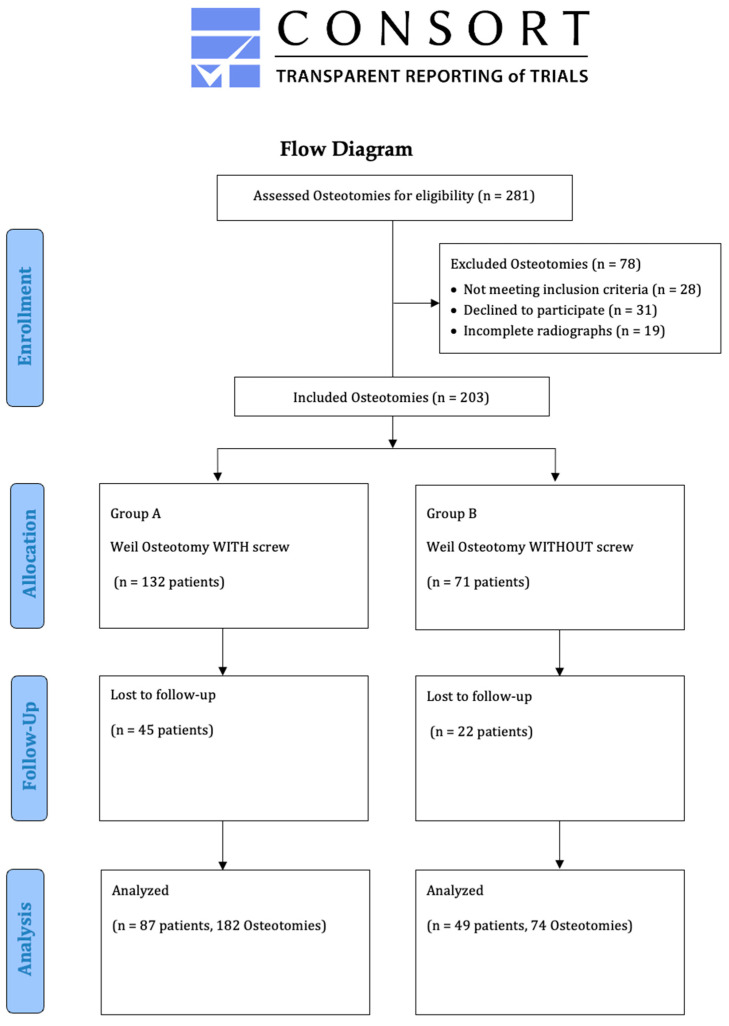
Study flow chart.

The radiological data of 136 feet of 127 patients with 256 Weil osteotomies between 2016 and 2021 were retrospectively enrolled in this monocentric comparative study (male feet, 17 (12.50%); female feet, 119 (87.50%); mean age: 63 years (range: 26–88 years)). The demographic characteristics of both groups were equally distributed (Table 1). All patients underwent isolated bony correction to restore a harmonious parabola of the forefoot. No additional soft tissue balancing procedures, such as a planar plate repair, were performed. For the purposes of this study, the patients were divided into two groups, as follows: group A included 182 Weil osteotomies with screw fixation, and group B included 74 osteotomies without screw fixation. The predominant underlying diagnoses were transfer metatarsalgia, but symptomatic claw toe deformity could also justify surgery. After a mean follow-up of 3.6 months (range: 1.2–17.0, standard error of the mean (SEM): 0.35, *p* = 0.138), the radiographic results were compared with the intraoperative and preoperative radiographs (Figure 3). All procedures were carried out in accordance with the 1964 Declaration of Helsinki. Subsequent amendments to the declaration were also acknowledged. The Ethics Committee thoroughly reviewed and approved this study prior to initiation (DRKS00031003). All persons involved in this study were instructed in compliance with the ethical guidelines.

### 2.2. Inclusion and Exclusion Criteria

Only radiographic findings of patients treated in the study center with a minimum age of 18 years were evaluated. There was no maximum age limit. Written informed consent was required prior to participation. Surgeries performed outside of the study center were not included. Operations due to septic or aseptic bone necrosis, underlying rheumatic disease, and traumatic dislocation of the metatarsophalangeal (MTP) joints of the small toes were excluded. Even Weil osteotomies as a revision procedure of nonunion and patients on acute or chronic pain therapy were also excluded. Both preoperative and postoperative radiographs had to be taken with weight-bearing.

### 2.3. Surgical Procedure and Rehabilitation Protocol

All patients underwent the same surgical procedure, whereby all Weil osteotomies of group A were performed with subsequent screw fixation by flat-cut technique, and all Weil osteotomies of group B were performed without screw fixation by wedge-cut technique with bone saw. All patients received an open approach. The average length of surgical incision was 3.5 cm. We always used longitudinal incisions between the metatarsals to address one or two metatarsals with one approach. If, for example, a Weil osteotomy is to be performed on metatarsals one to three, two separate incisions are made:one approach between the second and third metatarsal and another between the third and fourth metatarsal. The Weil osteotomy should be performed approximately parallel to the ground of the weight-bearing foot. The cut is then performed at the edge or 1–2 mm underneath the dorsal articular cartilage. The metatarsal head is then manually moved proximally by the desired distance. If the correction does not occur spontaneously, further soft tissue release may be necessary. The procedure can be considered successful if the desired straight position of the affected lesser toe is achieved and the joint space is visible in the dorsoplantar view. The procedure described up to this point was applied equally to all patients. If the Weil osteotomy was performed by the wedge-cut technique, a second cut 1–3 mm proximally followed, with removal of the bony wedge. Patients in group A subsequently received screw fixation of the metatarsal head. As a rule, screws with a diameter of 2 mm were used.

**Figure 4:**
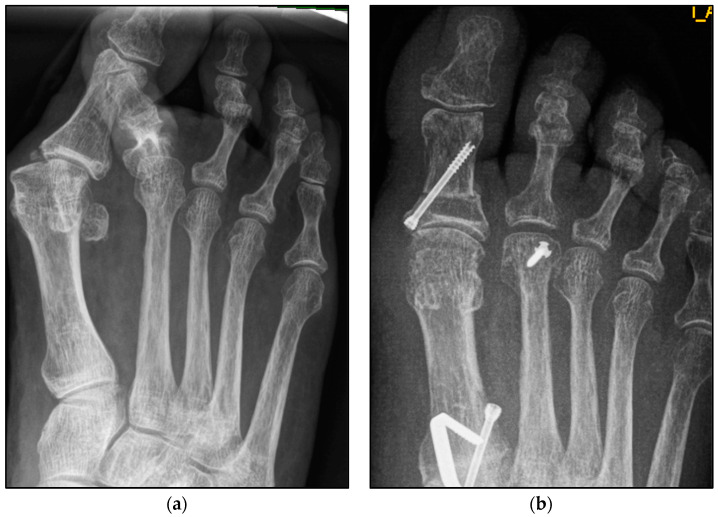
Pre- and postoperative radiographic results of combined hallux valgus surgery and Weil osteotomy of metatarsal 2 (group A with screw fixation), right foot. (**a**) Weight-bearing radiograph: anteroposterior view preoperative, (**b**) weight-bearing radiograph: anteroposterior view, 3 months postoperative.

Three equally experienced surgeons were involved in this study, and the operations were performed equally between them (Figure 3 and Figure 4).

After wound healing, regularly and 2 to 3 weeks postoperatively, pain-adapted full weight-bearing was permitted. A bandage or forefoot-offloading shoe was worn for 6 weeks on crutches. If necessary, depending on additional surgery, for example, hallux surgery of the first metatarsal, a longer period without weight-bearing had to be observed. Regardless of whether screw fixation was performed, the toes could and should be actively moved immediately after surgery.

### 2.4. Assessment Methods

Of interest was the restoration of the harmonious parabola of the forefoot and the metatarsophalangeal angle pre- and postoperative in the dorsoplantar view. Metatarsophalangeal joints 2–4 were included in the measurement. The metatarsophalangeal angle was determined using an axis through the metatarsal and the proximal phalanx of the affected toe. The mean lateral and medial deviation were evaluated in detail. Likewise, their difference was recorded, based on pre- and postoperative weight-bearing radiographs in the dorsoplantar view. All radiological measurements shown in the results section were taken by three surgeons and an independent radiologist. In addition, the correction potential and any bony malunion, nonunion, loosening of metal or failure of osteosynthesis, and relevant periarticular calcifications were evaluated.

Another evaluation criterion was based on the question of whether the affected MTP joint space was visible after the Weil osteotomy. A pre- and postoperative comparison of the radiographs was made, as well as an intergroup comparison. In addition, demographic data including age; sex; preexisting conditions such as rheumatism, arterial hypertension, diabetes mellitus, and BMI (associated with metabolic syndrome); and nicotine abuse were recorded for all patients.

### 2.5. Statistical Analysis

The primary objective of the present study was to demonstrate noninferiority of the Weil osteotomy without screw fixation versus with screw fixation at a mean follow-up of 3.6 months based on weight-bearing dorsoplantar foot radiographs. Other studies investigating a similar research question were consulted for the planning of this study. It should be noted that the studies analyzed for comparison included a significantly smaller number of patients. This fact strengthens the relevance of the data presented [5,6,7,8]. All statistical analyses were carried out using SPSS v. 23 software (IBM Dtl. GmbH, Ehningen, Germany). In addition, explorative and descriptive analyses for the queried evaluations and measurements of the pre- and postoperative radiographic findings (including within-group means, minima and maxima, medians, and standard deviations) were also applied. Furthermore, ANOVA and Student’s *t*-test were used. The power of this study was 0.8. The significance level was set to *p* < 0.05, and with it, there was a 95% confidence interval.

## 3. Results

**Figure 5:**
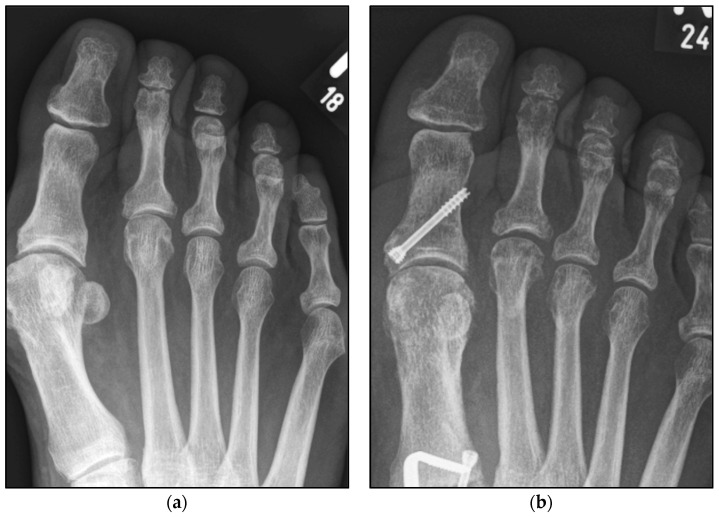
Pre- and postoperative radiographic results of combined hallux valgus surgery and Weil osteotomy of metatarsal 2 and 3 (group B without screw), right foot. (**a**) Weight-bearing radiograph: anteroposterior view preoperative, (**b**) weight-bearing radiograph: anteroposterior view, 3 months postoperative.

We collected the following data from the weight-bearing dorsoplantar foot radiographs after a mean follow-up period of 16 weeks (3.6 months). With no significant difference between groups, the mean MTP angle changed from 9.24° to 12.99° preoperative to postoperative (medial deviation: −n, lateral deviation: +n). As can be seen from the mean SEM in Table 2 and Figure 4 and Figure 5, the maximum medial and lateral deviations from pre- to postoperative were significantly different. The mean MTP only changed from 9.3 to 12.9 and was in the range of a mild lateral deviation. This was also observed equally in both groups (*p* > 0.05). Of note, the postoperative visibility of the joint space was documented significantly more often in the group without screw fixation; this means the space between the subchondral bone of the metatarsal and the toe was seen without overlay (*p* < 0.05).

In more than 70% of all cases (group A: 66.7%; group B: 81.4%), simultaneous hallux surgery was performed. Five delayed bony unions were documented after the Weil osteotomy, all in the group with screw fixation. No particular complaints from the patients concerned could be derived from the delayed union. The permanent nonunion was not seen in any of the patients. The radiographic results of the two techniques (wedge-cut or flat-cut technique) did not differ considerably; hence, a separate presentation of the results was omitted.

## 4. Discussion

For its part, the present study confirms that in addition to hammer toe malalignment, lateral and medial deviations of the lesser toes can also be successfully treated with Weil osteotomy. Weil osteotomy with screw fixation was the standard procedure in the hope of being able to make major corrections and avoid delayed union and nonunion. The results of the present study, in turn, relativize the necessity of screw fixation and confirm this with objectifiable data based on X-ray images. The most important finding for the authors is, therefore, that Weil osteotomy with and without screw fixation is an effective method to correct a malalignment of the forefoot. Even without screw fixation, the restoration of a harmonious metatarsal parabola was achieved, as originally aimed for with conventional Weil osteotomy with screw fixation [1,9]. Previous studies demonstrated the equivalence of patient satisfaction after Weil osteotomy with and without screw fixation [5,10,11]. Minimally invasive techniques, in particular, have become established [12].

Distal metatarsal minimal-invasive osteotomy (DMMO), therefore, appears to be particularly suitable for restoring a disturbed alignment of the forefoot to a harmonious parabola. Biz et al. also propagated this procedure. In the associated prospective study, they also examined the clinical and radiological results in accordance with the Maestro criteria [13]. The Maestro criteria use the length ratio between the metatarsals to assess whether a harmonious forefoot morphotype is present. From this, a harmonic forefoot morphotype is characterized by a so-called SM4 line that originates from the lateral sesamoid and passes through the head of the fourth metatarsal. The loss of length from the second to the fifth metatarsal is associated with a geometric progression of factor two [14]. Maestro et al. essentially derived four types from this, as follows: harmonic, M2M3 long, M4M5 hypoplasia, and M1 long [15]. In the present study, the morphotype M2M3 long, assessed on the basis of the preoperative radiographs, was represented with an above-average frequency of more than 30%. It is noteworthy that only about 30% of all asymptomatic feet are associated with a harmonious radiograph [15]. Biz et al. were also unable to demonstrate a correlation between the restoration of a harmonic alignment and an improved clinical outcome [13]. For the reasons mentioned previously, the Maestro criteria were not applied in this radiological study.

The present study even goes one step further. Firstly, it can be deduced from the results that screw fixation does not appear to be absolutely necessary. Secondly, a comparable correction can be achieved with and without screw fixation. All lateral and medial deviations of the lesser toes can apparently be corrected equally. The harmonious parabola of the forefoot restored intraoperatively remains the same after the bony union. This was shown by the weight-bearing radiographs. And thirdly, the authors were unable to recognize any difference between the procedures performed by the flat-cut technique and those performed by the wedge-cut technique. The authors see their assumption confirmed in the current literature and also assume that the decision in favor of one or the other technique is mainly subject to the preference of the respective surgeon and their experience [16,17]. Nonetheless, it should be noted that no conclusions can be drawn about clinical outcomes from the available radiological data. It is merely an observation that the technology had no obvious influence. However, the data appear to be quite suitable as a basis for a prospective comparison with questions to this effect.

The present evaluation was primarily devoted to the radiographic results. A radiographically confirmed delay or permanent nonunion was not documented in any of our cases, as seen in the minimally invasive procedure compared to the conventional Weil osteotomy in a study by Rivero-Santana et al. [18]. In contrast, the studies by Vandeputte et al. from 2000 and García-Ray et al. from 2004 already showed that Weil osteotomies, which were regularly performed open at that time, healed completely without nonunion [19,20]. A review by Pascual Huerta reads as follows: “Most of these techniques were based in the execution of one or more highly unstable metatarsal osteotomies without fixation followed by immediate weightbearing and whose results in the elevation or shortening of the metatarsal heads were often unpredictable” [21]. In particular, the authors of the present study see the statement that osteotomies without osteosynthesis lead to an unpredictable result refuted.

This assumption already calls into question the need for screw fixation, as even nonrelevant displaced metatarsal neck fractures can be conservatively treated [22]. So why should a flat saw cut have a higher potential for delayed union or nonunion? At least, there seems to be a consensus in the current literature that only grossly dislocated and unstable fractures of the metatarsal distal shaft or neck should be treated surgically [22,23]. Proximal fractures must be considered separately.

**Figure 6:**
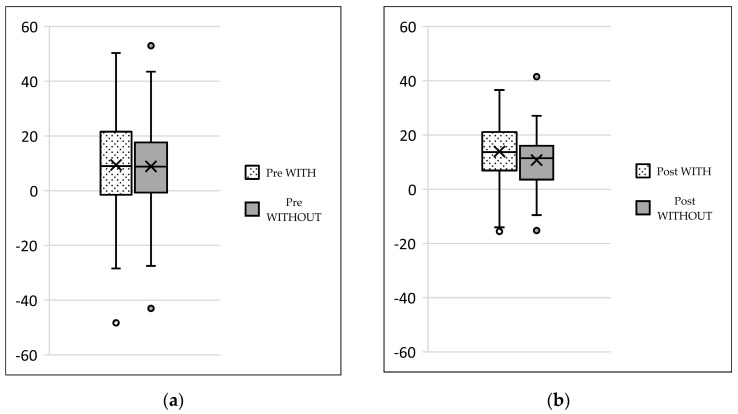
Pre- (**a**) and postoperative (**b**) toe deviation; medial (−)/lateral (+).

**Figure 7:**
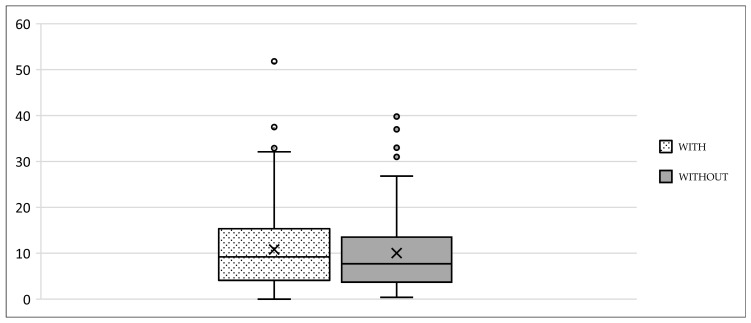
Extent of metatarsophalangeal angle correction, with and without screw fixation.

The underlying assumption of our study and the knowledge gained from our results can be further elaborated upon. An inharmonious transverse plane alignment can also be corrected with Weil osteotomy and does not require osteosynthetic fixation of the displaced metatarsal head [24]. The results confirm, based on postoperative weight-bearing radiographs, that the surgically induced correction is maintained regardless of screw fixation. An objectifiable criterion was the metatarsophalangeal angle measured pre- and postoperatively using weight-bearing radiographs in the dorsoplantar view (Figure 1 and Figure 2). In both treatment groups, it was possible to bring the maximum axis deviation closer to the mean value; the mean difference between pre- and postoperative metatarsophalangeal angle was only 3.8°. On average, 10.6 degrees were required to achieve the desired surgical result, regardless of screw fixation (Figure 4 and Figure 5). However, the significantly higher possibility of viewing the affected metatarsal joint space postoperatively can also be considered a successful outcome of the restoration without screw fixation. The visibility of the articular surface, in turn, proves the centering of the metatarsal head in the transverse plane as well as a sufficient correction in the sagittal plane (Figure 6 and Figure 7) [25]. Again, the inability to view the joint space indicates that inadequate correction of the claw toe deformity as well as the metatarsal, which was considered “too long”, was performed [1,14,26].

Moderate deviation of the lesser toes with increasing age is considered physiological [27,28,29]. The second ray, in particular, is affected by deformities such as hammer toe deformity. Despite the frequency of such a malalignment, it remains a significant impairment for the person concerned in everyday life. The extent of the deformity is described using many largely imprecise terms such as “mild”, “moderate”, and “significant”. An objectifiable extent cannot be derived from this. Despite all the possible inaccuracies that can also occur when taking X-rays, it remains the only objectifiable diagnostic tool that is almost always available, both in the description of the existing deformity and for monitoring success after the correction has taken place. To rule out the influence of an underlying common foot deformity, the varus, valgus, and splay foot components were also assessed. The mean talo-first metatarsal angle, mean talonavicular coverage angle, intermetatarsal angle 1–2, and intermetatarsal angle 1–5 in the dorsoplantar view were measured pre- and postoperatively, with no significant difference observed between the groups with and without screw fixation (*p* > 0.05). In accordance with the aim of this study, the preoperative values were not presented separately (Table 2).

The aforementioned achievements of Weil osteotomy without screw fixation have so far only been attributed to the modified Weil osteotomy with a minimally invasive procedure, the DMMO, based on studies with a significantly smaller number of cases [7]. However, it must be noted that DMMO requires a high learning curve [4,7,8]. In contrast, the authors of the present study postulate that Weil osteotomy without screw fixation does not involve an unusually high learning curve [8]. Rather, it is the less rigid treatment option that compensates for minor variations in the procedure. It should be noted that it is by no means an innovation in foot surgery to dispense with screw fixation in the context of osteotomies. Chevron osteotomy, for example, is a widely used distal diaphyseal osteotomy of the first metatarsal that is used to treat hallux valgus deformities. The first descriptions of this procedure did not include internal fixation [30]. Only recently, with the introduction of innovative implants such as double-threaded compression screws, has the discussion as to whether screw fixation is necessary at all become more important again. In addition, numerical meta-analyses also confirm that DMMO has a similar risk profile to conventional Weil osteotomy, with bony healing failure accounting for only 3%. Nonunion did not occur at all in the present study.

The following limitations of this study should be mentioned. This was a monocentric study with a retrospective design. The choice of procedure, Weil osteotomy with or without screw fixation, was largely determined by the experience of the respective surgeon. In addition, the evaluation criterion, “visibility of the joint space,” is subject to relatively low reliability. Due to the lack of sample size analysis in the retrospective study presented here, the actual power of the results could be reduced. This is common to all comparable studies. The follow-up varied between 1.2 and 17 months. A loss of correction, especially in the early postoperative phase, would be conceivable, although this was not detected in the radiological control under full weight-bearing.

Another inaccuracy in the measurement of the MTP angle could be due to a claw/hammer toe deformity not detected in the dorsoplantar view. However, this bias would be common to all 256 Weil osteotomies.

## 5. Conclusions

In the absence of bony malunion and the satisfactory restoration of a harmonious parabola of the forefoot, in the present study, Weil osteotomies with and without screw fixation are considered apparently equivalent based on dorsoplantar radiographs. Based on the available data, with similar corrective potential, there does not appear to be a need for regular screw fixation.

## Figures and Tables

**Table 1:** Patient characteristics, n = 136 feet, n = 127 patients.

Characteristic		With Screw Fixation (n = 87)	Without Screw Fixation (n = 49)	All (n = 136/127)	*p*
Age, years	Mean	63.44	63.02	63.42	0.955
	SEM	1.36	1.46	1.02	
	Minimum	26.00	36.00	26.00	
	Maximum	88.00	83.00	88.00	
BMI, kg/m^2^	Mean	26.72	25.65	26.33	0.183
	SEM	0.55	0.67	0.431	
	Minimum	17.78	19.30	17.78	
	Maximum	42.20	39.20	42.20	
Sex, n (%)	Male	12 (13.79)	5 (10.20)	17 (12.50)	0.043
	Female	75 (86.21)	44 (89.89)	119 (87.50)	
Affected side, n (%)	Left	36 (45.57)	27 (56.25)	63 (49.61)	0.101
	Right	35 (44.30)	20 (41.67)	55 (43.31)	
	Both sides	8 (10.13)	1 (2.08)	9 (7.08)	
Smoker, n (%)	Yes	12 (13.79)	6 (12.24)	18 (13.23)	0.767
	No	75 (86.21)	43 (87.76)	118 (86.76)	
Preexisting conditions (multiple answers), n (%)	Metabolic syndrome-associated	35 (40.23)	20 (40,82)	55 (40.44)	0.681
	rheumatism	4 (4.59)	1 (2.04)	5 (3.67)	
	Others	47 (54.02)	28 (57.14)	75 (55.15)	
	None	15 (17.24)	7 (14.29)	22 (16.18)	

BMI, body mass index; SEM, standard error of the mean.

**Table 2:** Radiographic outcome according to treatment, based on the number of osteotomies.

Measurement		With Screw Fixation (n = 182)	Without Screw Fixation (n = 74)	All (n = 256)	*p*
MTP angle, preoperative	Mean	9.42	8.81	9.24	0.847
	SEM	1.36	2.06	1.13	
	Minimum *	−48.30	−43.00	−48.30	
	Maximum **	50.30	55.00	55.00	
MTP angle, postoperative	Mean	13.91	10.75	12.99	0.022
	SEM	0.75	1.12	0.63	
	Minimum *	−15.50	−15.52	−15.50	
	Maximum **	36.60	41.50	41.50	
Extent of angle correction	Mean	10.81	10.02	10.58	0.510
	SEM	0.65	1.00	0.55	
	Minimum	0.00	0.40	0.40	
	Maximum	51.80	39.80	51.80	
Visibility of MTP joint space given, preoperative (%)	Yes	93 (51.09)	41 (55.41)	134 (52.34)	0.619
	No	86 (47.25)	33 (44.59)	119 (46.48)	
Visibility of MTP joint space given, postoperative (%)	Yes	155 (85.17)	71 (95.95)	226 (88.28)	0.028
	No	24 (13.19)	3 (4.05)	27 (10.55)	
	n.a.	3 (1.64)	0 (0.00)	3 (1.17)	
Talo-first metatarsal angle, postoperative	Mean	11.04	9.17	10.36	0.130
	SEM	0.73	0.99	0.59	
Talonavicular coverage angle, postoperative	Mean	10.81	8.24	9.87	0.055
	SEM	0.83	0.99	0.65	
Hallux valgus angle, postoperative	Mean	14.06	13.45	13.84	0.618
	SEM	0.72	1.04	0.59	
Intermetatarsal M1–M5A, postoperative	Mean	24.00	25.46	24.53	0.147
	SEM	0.58	0.84	0.48	

SEM, standard error of the mean; MTP, metatarsophalangeal joint; MT, metatarsal; M1–M5A, angle between the first and fifth metatarsals; *, maximum medial deviation; **, maximum lateral deviation.

## Data Availability

All data intended for publication are included in the manuscript.
